# Adipocyte Oncostatin Receptor Regulates Adipose Tissue Homeostasis and Inflammation

**DOI:** 10.3389/fimmu.2020.612013

**Published:** 2021-03-29

**Authors:** David Sanchez-Infantes, Jacqueline M. Stephens

**Affiliations:** ^1^ Department of Endocrinology and Nutrition, Germans Trias i Pujol Research Institute, Barcelona, Spain; ^2^ Department of Basic Sciences of Health, Area of Biochemistry and Molecular Biology, Universidad Rey Juan Carlos, Alcorcon, Spain; ^3^ Department of Biological Sciences and Pennington Biomedical Research Center, Louisiana State University, Baton Rouge, LA, United States

**Keywords:** adipocyte, OSM, Inflammation, OSM receptor, fat, adipose tissue, insulin resistance

## Abstract

Adipocytes are the largest cell type in terms of volume, but not number, in adipose tissue. Adipocytes are prominent contributors to systemic metabolic health. Obesity, defined by excess adipose tissue (AT), is recognized as a low-grade chronic inflammatory state. Cytokines are inflammatory mediators that are produced in adipose tissue (AT) and function in both AT homeostatic as well as pathological conditions. AT inflammation is associated with systemic metabolic dysfunction and obesity-associated infiltration and proliferation of immune cells occurs in a variety of fat depots in mice and humans. AT immune cells secrete a variety of chemokines and cytokines that act in a paracrine manner on adjacent adipocytes. TNFα, IL-6, and MCP-1, are well studied mediators of AT inflammation. Oncostatin M (OSM) is another proinflammatory cytokine that is elevated in AT in human obesity, and its specific receptor (OSMRβ) is also induced in conditions of obesity and insulin resistance. OSM production and paracrine signaling in AT regulates adipogenesis and the functions of AT. This review summarizes the roles of the oncostatin M receptor (OSMRβ) as a modulator of adipocyte development and function its contributions to immunological adaptations in AT in metabolic disease states.

## Introduction

The global obesity rate has nearly doubled since 1980 (1). This high incidence poses a massive economic burden on healthcare systems. More importantly, obesity is frequently accompanied by adverse metabolic effects including hypertension, dyslipidemia, fatty liver, insulin resistance and type 2 diabetes (T2D) (2). In addition, obesity (3) and T2DM (4) are prominent risk factors for the severity of COVID-19 infections. Although obesity is a threat to global health, treatment options remain limited, and they are often ineffective or invasive (*e.g.* bariatric surgery) (5).

Obesity occurs when energy intake exceeds energy expenditure, but this relationship is complex, as many factors influence these two parameters. Positive energy balance causes WAT to expand by adipocyte hyperplasia, hypertrophy, or a combination of these processes. In addition to lipid storage, adipocytes have important endocrine functions whereby they secrete hormones (leptin, adiponectin, etc.), microRNAs, exosomes, and lipids that contribute to systemic metabolic health (6). There is evidence that the release of proinflammatory cytokines, such as Tumor Necrosis Factor α (TNFα) and Monocyte chemoattractant protein 1 (MCP-1) that can occur in obesity is driven by stress responses related to WAT expansion, although specific mechanisms involved remain to be elucidated (7).

In addition to adipocytes, there are several other cell types within WAT, including different types of macrophages and T cells. The non-adipocyte cells in AT, such as immune, endothelial, perivascular, and stromal cells, as well as preadipocytes, collectively comprise the stromal vascular fraction (SVF). The cell numbers of the SVF are greater than number of adipocytes in white adipose tissue depots. Obesity is associated with changes in the relative abundance and activation states of various immune cell subpopulations in AT, as well as with altered endocrine properties of adipocytes themselves. Many of the proinflammatory cytokines produced in AT act in a paracrine manner and typically do not contribute to circulating levels of these signaling mediators. Proinflammatory cytokines made in AT can inhibit adipocyte differentiation and induce insulin resistance in adipocytes, and modulation of both these processes in AT has systemic effects (8–10). Although less studied than other AT cytokines, OSM clearly contributes to AT homeostasis (11–13), and increased OSM levels in AT promote systemic metabolic dysfunction through effects on both adipocyte development and adipose tissue function.

## OSM and Its Specific Receptor OSMRß: Source and Biology

The gp130, or interleukin (IL)-6, family is a group of structurally similar cytokines that includes IL-6, IL-11, IL-27, neuropoietin, leukemia inhibitory factor (LIF), OSM, cardiotrophin-1, ciliary neurotrophic factor, and novel neurotrophin-1/B cell stimulating factor-3 or cardiotrophin-like cytokine (14). These cytokines regulate a variety of complex biological processes, including hematopoiesis, immune responses, inflammation, stem cell potency, mammalian reproduction, cardiovascular action, and neuronal survival (15). Also, gp130 cytokines have been proposed as potential therapeutic targets for obesity treatment (16). Hence, there is a strong rationale for studying gp130 cytokines in modulating metabolic processes in WAT and other tissues involved in obesity and related diseases.

All members of the IL-6 cytokine family require glycoprotein 130 (gp130) as a common signal transducer in their receptor complexes. Unlike other gp130 cytokines, OSM has its own specific receptor (OSMRβ) that heterodimerizes with gp130 but is not used by other gp130 cytokines (17) and mediates the majority of OSM effects. OSM and LIF evolved by gene duplication relatively recently (18), and they share substantial sequence identity (19). Though originally identified for its ability to inhibit cancer growth in humans (20), OSM can modulate a variety of other biological processes, including liver development and regeneration (21, 22), hepatic insulin resistance and steatosis (23), inflammation (24), and cardiomyocyte dedifferentiation and remodeling (25). There is some evidence that OSM is the only gp130 cytokine with the unique ability to signal through two distinct receptor units-the gp130/LIFR (26) and the gp130/OSMRβ complex (17). However, other studies have shown that murine OSM signals only through the gp130/OSMRβ receptor complex (27–29).

OSM is produced by activated T cells and macrophages (20, 30, 31), and elevated OSM levels are found in a variety of inflammatory diseases in humans, including inflammatory bowel disease, rheumatoid arthritis, cancer, and obesity (12, 32–35). Our own research has shown that OSM is present in the SVF of AT, but not in adipocytes (11). Purification of immune cells in AT revealed that T cells and macrophages were the main sources of OSM in adipose tissue in mice (36). Although OSM is produced in immune cells, the OSM receptor (OSMRß) is present in both adipocytes and immune cells (36). However, upregulation of OSMRß expression by high-fat diet is observed only in adipocytes (36).

## Effects of OSM-OSMRß Interaction in Pathological Conditions

The molecular signaling caused by OSM-OSMRß interaction has been suggested to modulate several inflammatory processes, including obesity-related insulin resistance (11, 13). One of several mechanisms involved in the ability of excess OSM to promote metabolic dysfunction is the control of adipogenesis. Inhibition of fat cell differentiation and adipose tissue expansion has been recognized as a causative factor for insulin resistance for over twenty years (37). Indeed, factors that inhibit adipogenesis, including OSM, tumor necrosis factor alpha and interferon gamma have been shown to have metabolically unfavorable effects such as insulin resistance (38). It is well established that OSM inhibits adipocyte development of both brown and white adipocytes *in vitro* (39–41). Mice with a global deletion of OSMRβ have increased adipose tissue mass (42), supporting the concept that OSM acts to inhibit adipocyte development and that lack of OSM signaling leads to increased AT expansion. There is also evidence to suggest that OSM treatment of mice reduces body weight and adiposity (42, 43). However, it should be noted that the OSM doses used in these mouse experiments were very high (12.5 ng/g body weight, administered twice daily) and may have caused indirect effects on fat mass. The anti-adipogenic effects of OSM have also been shown in human preadipocytes (13). In regard to the molecular mechanisms involved in the impairment of adipogenesis, OSM has been shown to inhibits C/EBPα and PPARγ (peroxisome proliferator-activated receptor γ) expression, two key transcription factors involved in adipogenesis (40, 44). In terms of modulation of lipid and glucose homeostasis, the anti-adipogenic effects of OSM could have systemic consequences. In addition to AT, the liver is an essential metabolic organ for lipogenesis, lipid uptake, and fatty acid b-oxidation and liver is responsive to OSM signaling (45). Some studies show that the OSMRβ expression levels negatively correlate with mRNA levels of gluconeogenic genes. Moreover, OSMRβ ablation lead to decreased levels of genes related to cholesterol efflux and fatty acid β-oxidation, and increased expression of genes that regulate cholesterol synthesis, fatty acid synthesis, and uptake (45). Hence, it is likely that OSM promotes inflammation and metabolic dysfunction at least in part by inhibiting the development of new adipocytes., but there is also evidence to show OSM also regulates lipid metabolism pathways in the liver.

**Figure 1:**
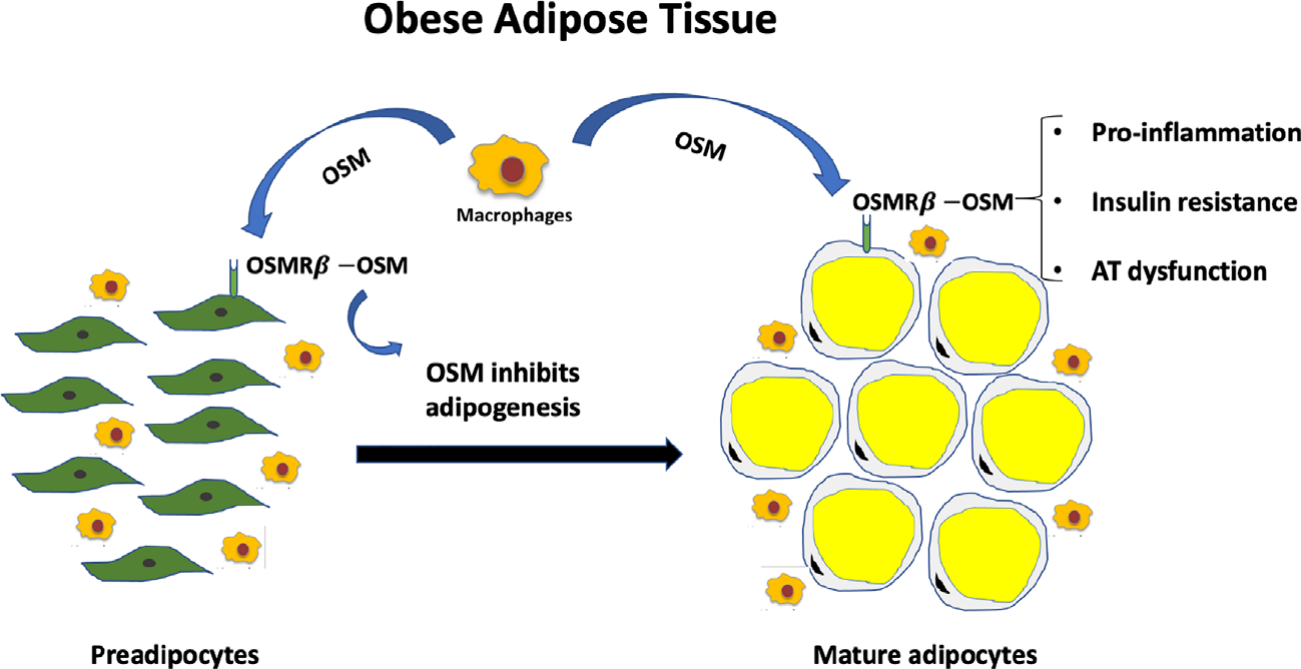
Excess OSM and lack of adipocyte OSM signaling contributes to metabolic dysfunction. Less than half of the cells that comprise white adipose tissue depots are adipocytes. OSM is not produced in adipocytes, but in adipose tissue macrophages in conditions of obesity. OSM acts on preadipocytes to inhibit adipogenesis and acts on mature adipocytes to promote inflammatory signaling and insulin resistance in adipocytes. Both a loss of OSM signaling in adipocytes or excess OSM in adipose tissue promote systemic metabolic dysfunction.

In addition to regulating adipocyte differentiation, OSM has been proposed to contribute to AT immune response. In contrast to IL-6 which is directly induced through the TLR-nuclear factor k-B pathway (46), OSM is secreted by activated macrophages through a PGE2-cyclic adenosine monophosphate- protein kinase A pathway (47, 48). In adipose tissue from obese mice, OSMRß has been reported to be increased in the SVF, especially in the F4/80-positive ATMs (adipose tissue macrophages), suggesting that OSM signaling is strongly associated with the pathogenesis of obesity and related metabolic disorders (43). OSM binding to OSMRß modulates inflammatory states, both *in vitro* and *in vivo*. Expression of stromal cell-derived factor 1 alpha (SDF-1α) has been reported to be suppressed by OSM treatment of adipocytes (49). SDF-1α, also known as CXCL12, regulates the trafficking of bone marrow progenitor cells, as well as the transendothelial migration of leukocytes (50, 51). Further studies are required to determine whether altered SDF-1 levels play a role in mediating OSM’s effects on homeostasis or metabolic dysfunction. In addition to SDF-1, there is evidence that plasminogen-activator inhibitor 1 (PAI1) is also directly regulated by OSM (11). The ability of OSM to induce PAI1 is dependent on OSMRß expression in cultured murine adipocytes (11). Although SDF-1 and PAI-1 may play a role in OSM function in AT, no rigorous studies have identified or directly evaluated OSM-regulated genes in adipocytes. Interestingly, *in vitro* experiments in brown adipocytes have demonstrated that OSM signaling *via* the OSMRß results in an increase in TNFα and MCP-1 (or C-C Motif Chemokine Ligand 2, Ccl2) mRNA levels, and interleukin 6 protein and each of these cytokines are involved in the recruitment and activation of macrophages in AT (13, 41). Therefore, it is reasonable to predict that in obesity, the overexpression of OSM by immune cells, including macrophages, is acting on adipocytes to induce the secretion of inflammatory cytokines that promote infiltration and activation of more macrophages. This vicious cycle leads to a low-grade chronic inflammatory state that contributes to the development of insulin resistance (**Figure 1**). Moreover, in humans with obesity, OSM levels correlate positively with inflammatory markers and negatively with glucose transporter 4 (Glut4), suggesting that signaling through OSMRß could promote an immunological response in AT that impairs glucose homeostasis (13, 41).

*In vivo* experiments have demonstrated that mice lacking OSMRβ, specifically in adipocytes, have significant increases in AT mass and OSM expression in fat, as well as enhanced adipose tissue inflammation, as compared to floxed littermate controls (36). The latter observation is unexpected, given that OSM signaling is known to promote inflammation. Although data from this study suggests that enhanced OSM-OSMRβ action in other AT cells, including immune populations, is consistent with the increased inflammatory immune response and insulin resistance phenotype in mice that lack OSM receptor specifically in adipocytes (36). Hence, by blocking OSM signaling in adipocytes *via* loss of the OSM receptor, the AT levels of OSM increase and promote metabolically unfavorable effects by acting on non-adipocyte cells present in AT.

One method to assess the importance of an endocrine mediator is to inhibit its activity with an immunoneutralization approach. Immunoneutralizing OSM is a complementary approach to knocking down the OSM receptor in adipocytes. In a recent study, we used high-fat fed C57BL/6J mice to induce OSM expression in AT and performed OSM immunoneutralization. Mice that received a specific anti-OSM antibody had improved inflammatory responses as compared to mice treated with a control IgG antibody (13). Moreover, OSM immunoneutralization normalized glucose levels and decreased expression of inflammatory genes in adipose tissue. However, OSM immunoneutralization did not significantly alter whole-body glucose tolerance or systemic insulin sensitivity (13). Although there are limitations with this approach, these studies underscore the need to understand the cell and tissue specific effects of both physiological and pathological functions of OSM.

In addition to its functions in AT and association with obesity and Type 2 diabetes, OSM has been shown to play a role in a variety of disease conditions. Several studies have identified the OSM-OSMRß interaction as a potential therapeutic strategy for several pathological conditions. The selective inhibition of OSM by a neutralizing antibody suggested that paracrine actions of OSM in mammary fat played a role in breast cancer progression (34). In addition, OSM has been identified as a potential biomarker and therapeutic target in inflammatory bowel disease (35). The ability to target OSM in inflammatory bowel disease is important as up to 40% of patients do not respond to anti-TNF agents. Of note, an anti-OSM monoclonal antibody has recently been shown to be well tolerated in healthy subjects, and has demonstrated sufficient affinity to achieve target engagement in systemic circulation and target skin tissue, supporting further clinical investigation of anti-OSM antibodies for inflammatory diseases (52).

## Conclusions

In summary, OSM is a member of a large cytokine family, but its unique functions in adipocytes drive its effects on metabolic health. Levels of OSM and its receptor are elevated in AT in conditions of obesity and insulin resistance in mice and man (12). The roles of OSM have been elucidated using a wide range of approaches including global and adipocyte-specific knockout of the OSM receptor, as well as immunoneutralization of OSM in metabolically compromised mice. In AT, elevated levels of immune cell-derived OSM act on adjacent AT cells to inhibit preadipocyte differentiation and to enhance proinflammatory responses in adipocytes. Although adipose tissue OSM levels correlate with systemic metabolic dysfunction, a loss of OSM receptor in adipocytes is also associated with impaired metabolic responses. This finding is consistent with a role for OSM signaling in healthy adipocytes and in AT homeostasis. Of note, there is a precedent for the contribution of inflammatory mediators in normal adipocyte function, as suppressing adipocyte inflammation impairs AT function and promotes insulin resistance (53, 54). Notably, the suppression of macrophage inflammation has little effect on obesity-induced insulin resistance, but inhibition of inflammatory signaling in adipocytes substantially effects systemic metabolic function (54). Inflammatory signaling in adipocytes plays a role in maintaining normal adipose tissue function and OSM signaling in adipocytes and adipose tissue is important for normal adipose tissue function and systemic metabolic health.

## Author Contributions

Both authors contributed equally to the preparation and editing of this review. All authors contributed to the article and approved the submitted versión.

## Funding

DS-I has been supported by grants CP15/00106 and FIS PI17/01455 from Carlos III National Institute of Health and European Regional Development Fund (ERDF).

## Conflict of Interest

The authors declare that the research was conducted in the absence of any commercial or financial relationships that could be construed as a potential conflict of interest.
